# Pedicled transverse rectus abdominis myocutaneous flap removal after 21 years: a case report

**DOI:** 10.1093/jscr/rjaf654

**Published:** 2025-09-21

**Authors:** Natasha Lim, Lee Chee Meng, Marissa Teo, Allen Wei-Jiat Wong

**Affiliations:** Sengkang General Hospital Singapore, Department of General Surgery, Singapore 544886, Singapore; Sengkang General Hospital Singapore, Department of Breast, Trauma, General Surgery, Singapore 544886, Singapore; Sengkang General Hospital Singapore, Department of Pathology, Singapore 544886, Singapore; Sengkang General Hospital Singapore, Plastic, Reconstructive and Aesthetic Service, Department of Surgery, Singapore 544886, Singapore

**Keywords:** pedicled transverse rectus abdominis myocutaneous flap, fatty attenuation of myocutaneous flaps, loss of muscle volume of myocutaneous flaps

## Abstract

Despite the widespread use of free abdominal based flaps for autologous breast reconstruction, the pedicled transverse rectus abdominis myocutaneous (p-TRAM) flap remains a popular alternative. Although the p-TRAM flap has been commonly utilized since the 90s, there is a paucity of information on the long-term changes that occur in the p-TRAM flap with the passage of time. Imaging studies have demonstrated changes in muscle volumes and fatty attenuation of myocutaneous flaps over time. However, the use of a histopathological approach in analysing the changes in a myocutaneous flap is rarely done as rarely do patients undergo a removal of a healthy flap. Our report seeks to show the loss of the muscle volume over time through a histopathological approach in a patient who underwent p-TRAM Flap removal after 21 years.

## Introduction

Despite the widespread use of free abdominal based flaps for autologous breast reconstruction, the pedicled transverse rectus abdominis myocutaneous (p-TRAM) flap remains a popular alternative for various reasons, including high risk aversion and/or lack of microsurgical expertise. This surgical option has the bonus of creating a natural fall appearance to the new breast mound and simultaneously performing a lipectomy of the abdomen [1]. Studies have shown that reconstruction with the p-TRAM generally produces good results and pleased patients [2].

Although the p-TRAM flap has been commonly utilized since the 90s, there is a paucity of information on the long-term changes that occur in the p-TRAM flap with the passage of time. Imaging studies have demonstrated changes in muscle volumes and fatty attenuation of myocutaneous flaps over time [3–7]. However, the use of a histopathological approach in analysing the changes in a myocutaneous flap is rarely done as rarely do patients undergo a removal of a healthy flap. In our report, we seek to show the loss of the muscle volume over time through a histopathological approach in a patient who underwent p-TRAM Flap removal after 21 years.

## Case presentation

**Figure 1:**
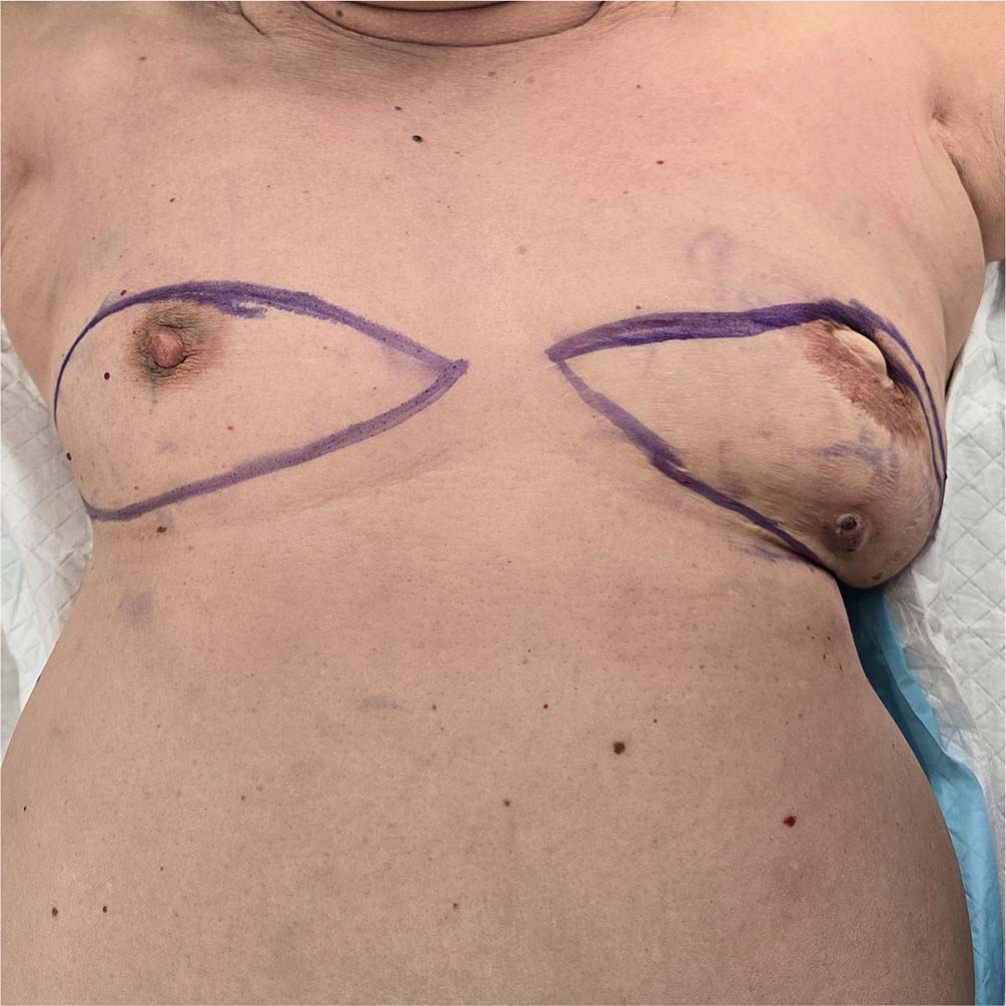
Preoperative picture of bilateral breasts.

**Figure 2:**
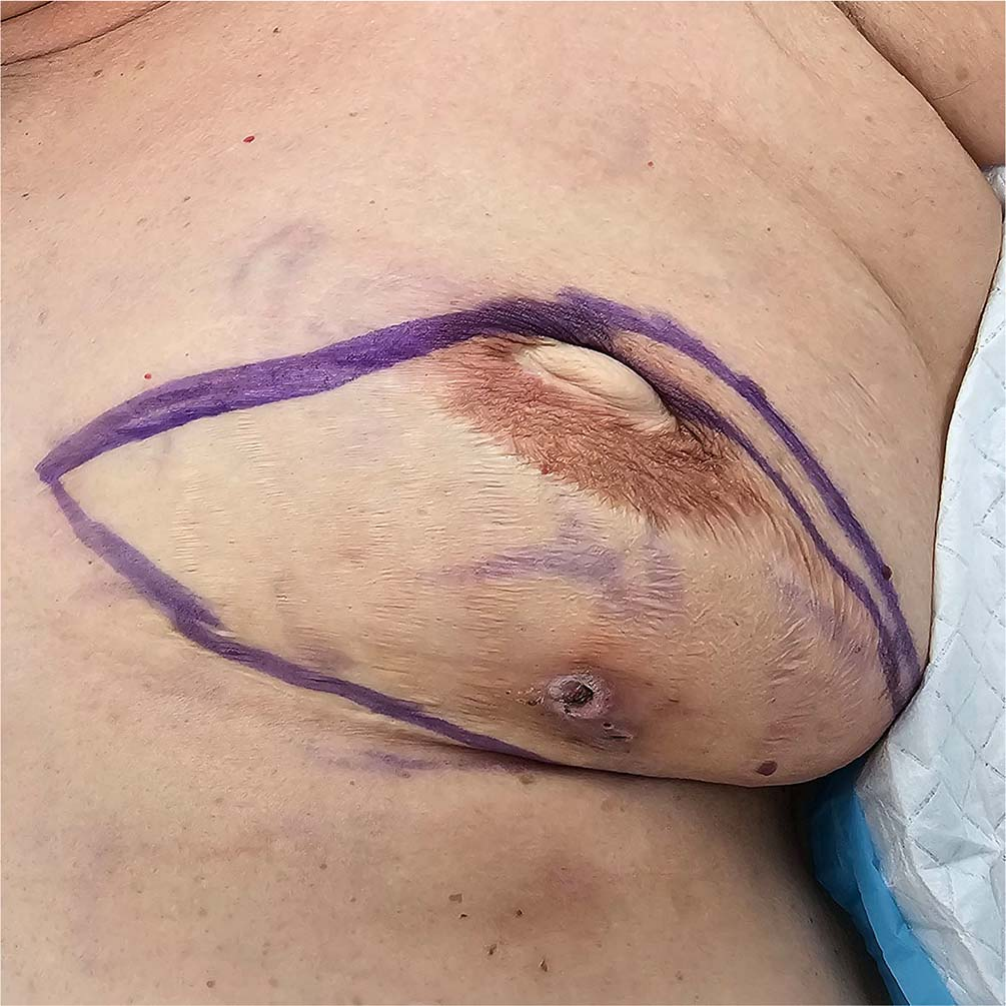
Preoperative picture of left breast.

In 2003, our 73 year old patient, was diagnosed with left breast cancer and underwent a left skin sparing mastectomy and pedicled transverse rectus abdominis myocutaneous flap reconstruction. She re-presented in breast clinic in July 2024 with an abscess in the reconstructed breast. Opportunistic screening with ultrasound and mammogram was performed for her right breast, resulting in an incidental finding of right breast solid papillary carcinoma. After discussion of resection and reconstruction options, she elected for a right breast mastectomy and requested for a removal of her left breast p-TRAM flap as well as she was keen to achieve a more symmetrical outcome (Figs 1 and 2).

**Figure 3:**
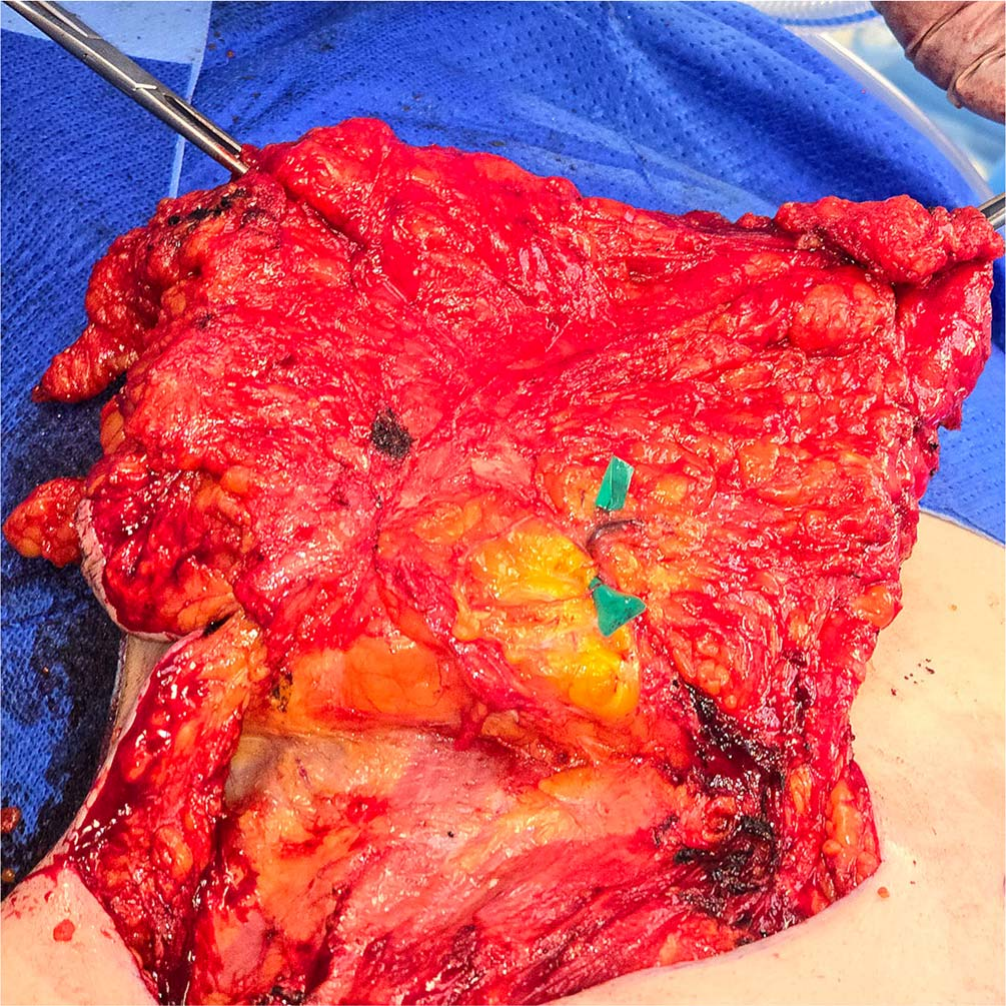
Transverse rectus abdominis myocutaneous (TRAM) flap.

**Figure 4:**
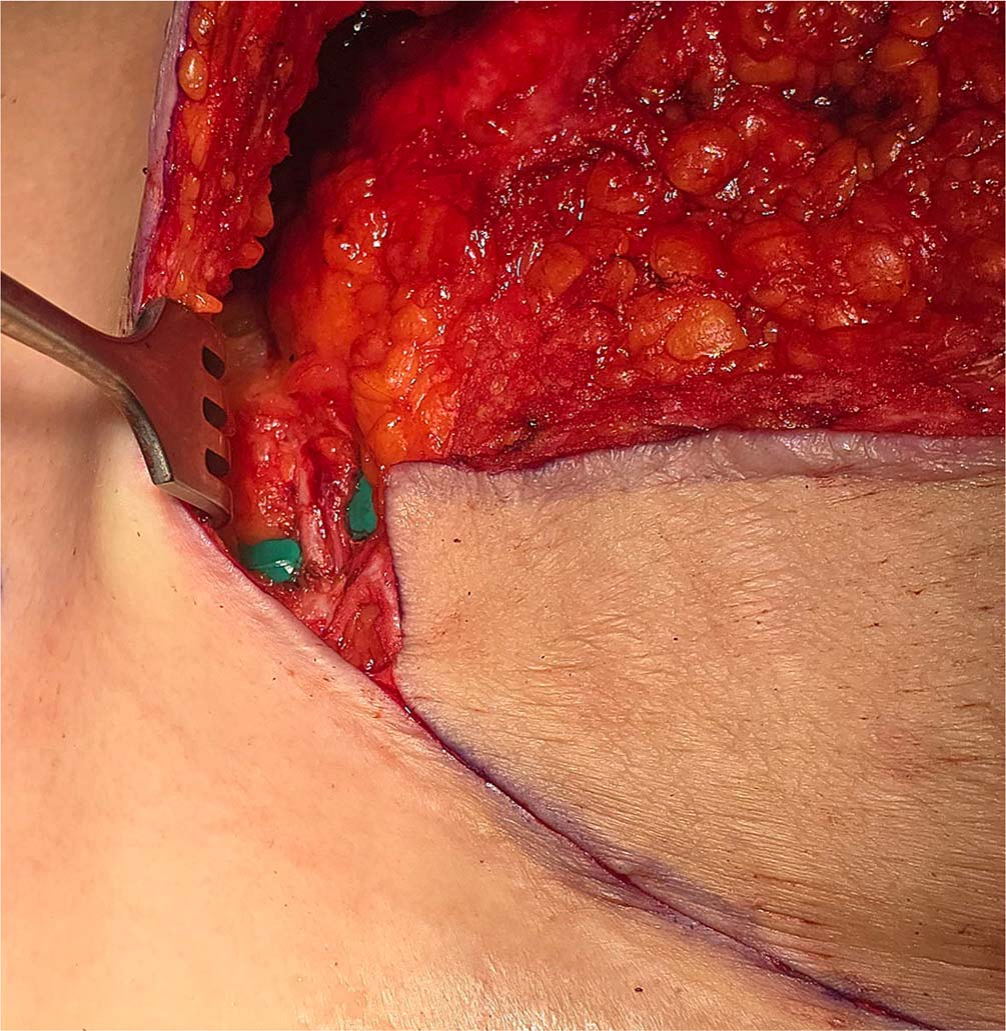
Superior epigastric paddle over the green contrast.

**Figure 5:**
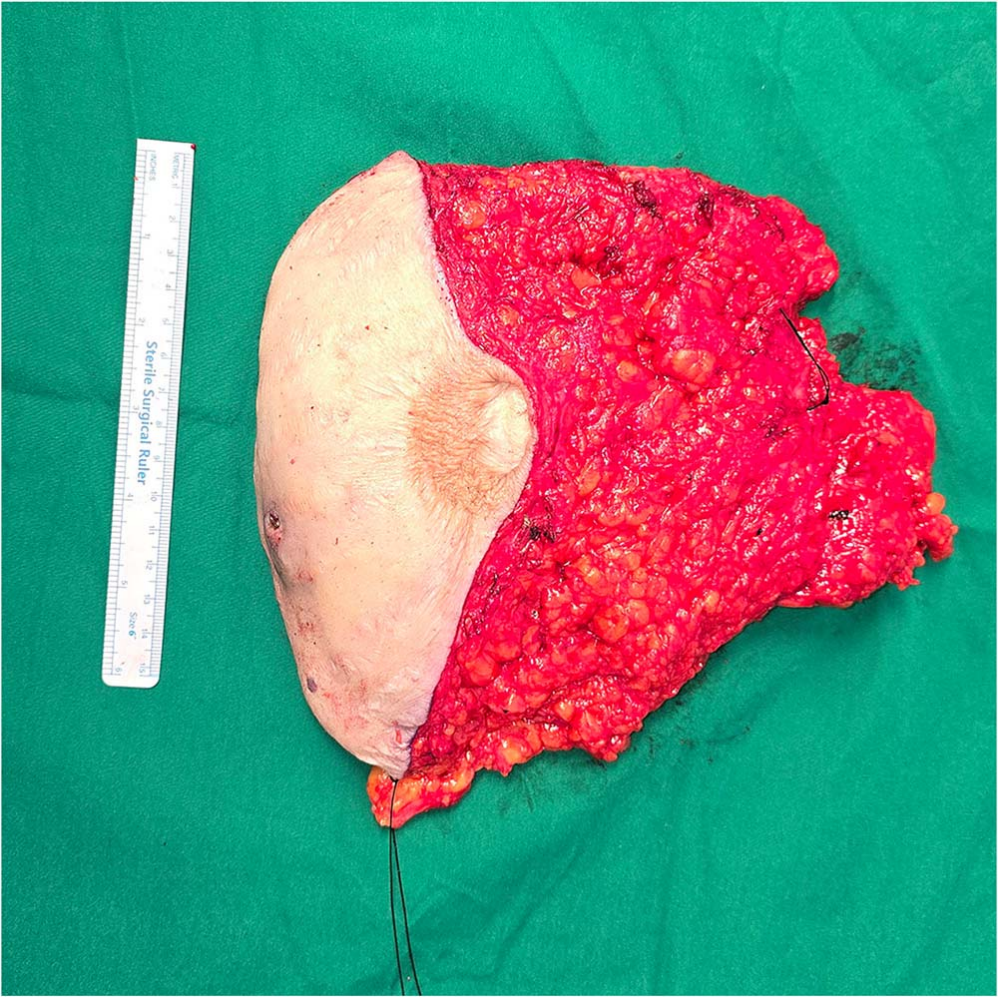
TRAM flap.

During explantation of the left-sided p-TRAM Flap, the rectus muscle was found to be attenuated and replaced with fibrous fatty tissue. The superior epigastric pedicle was still identifiable, with good pulsatile flow (Figs 3–5).

**Figure 6:**
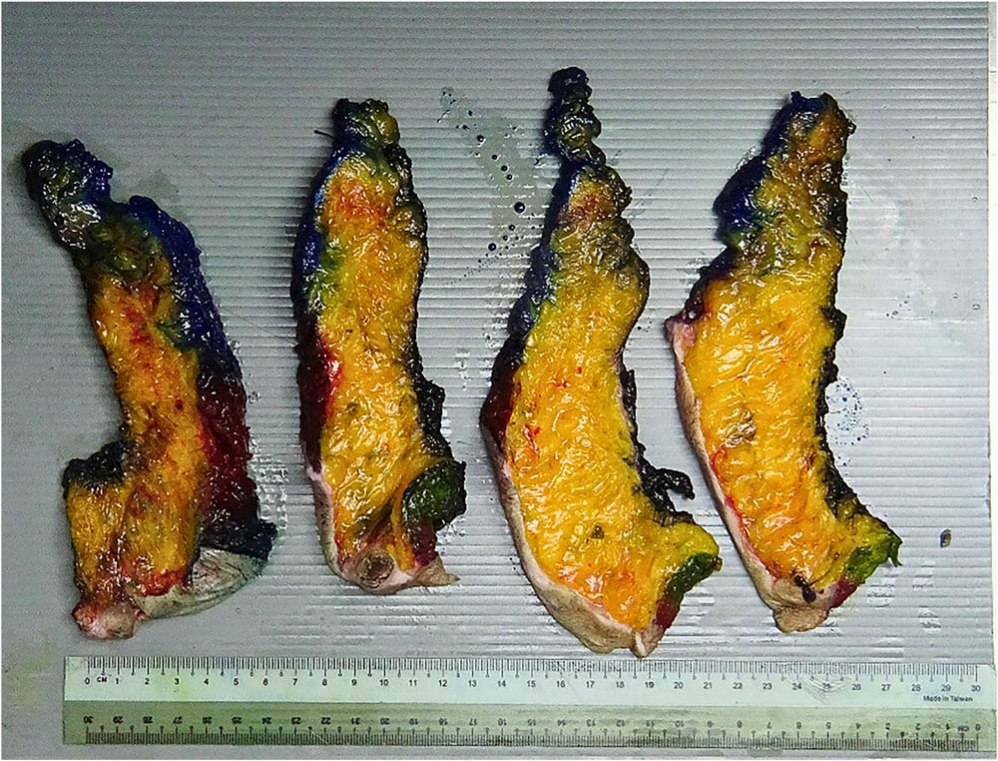
TRAM flap trimmed specimen, posterior margin labelled in black.

**Figure 7:**
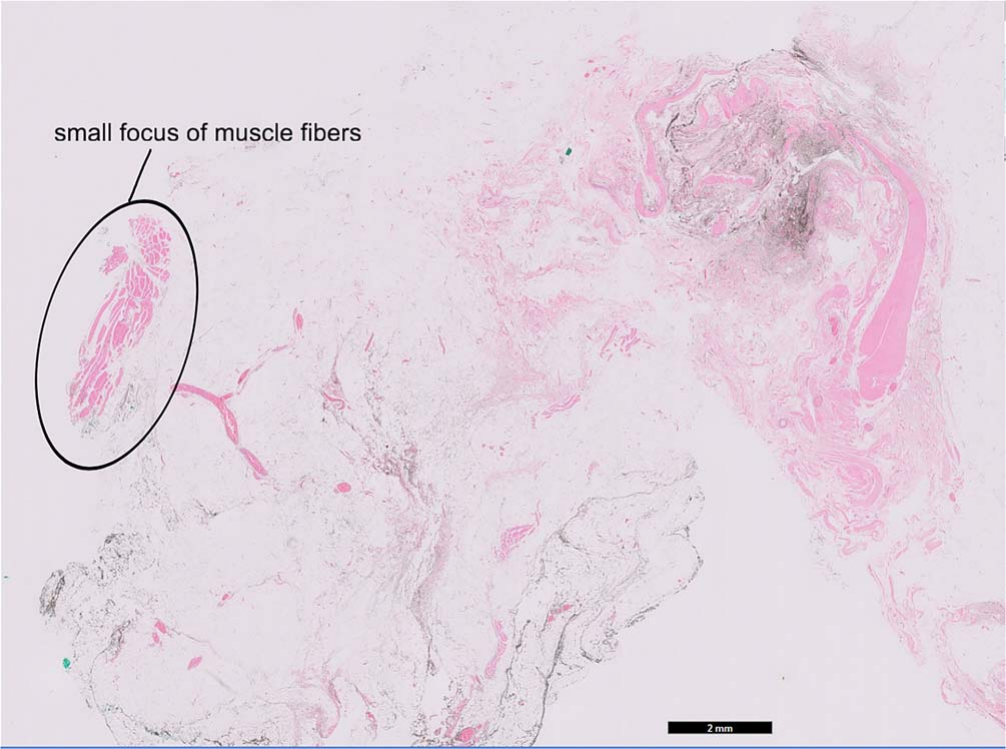
Hematoxylin and eosin slides.

The attenuation of the muscle was confirmed on histopathological analysis. The excised specimen was predominated by fatty tissue with no discernible muscle (Fig. 6) Haematoxylin and eosin staining showed a small focus of muscle fibres in the top left corner of the slide surrounded by predominant fat and blood vessels (Fig. 7).

## Discussion

This case demonstrates long-term fatty attenuation and muscle loss in a pedicled transverse rectus abdominis myocutaneous flap reconstruction over a 21-year period, confirmed through histopathological examination. These findings are consistent with prior studies reporting similar phenomena in myocutaneous flaps, as observed through imaging modalities.

Computed tomography (CT) imaging of patients who underwent TRAM flap reconstruction between 1991 and 1997 in Michigan predominantly revealed fatty attenuation within the flap. This contrasts with the irregular soft-tissue attenuation of fibroglandular tissue mixed with fat typically seen in the native breast. Additionally, the rectus muscle used in TRAM flap reconstruction was shown to decrease in thickness over time postoperatively, with the most significant changes occurring beyond the immediate post-surgical period, compared to the contralateral, native rectus muscle [3].

Mammographic evaluation of TRAM flaps conducted in Texas in 1990 also revealed a relatively homogeneous, predominantly fatty composition. The volume of the rectus abdominis muscle ranged from a well-defined bulky mass to faint, almost imperceptible strands of tissue [4].

Magnetic resonance imaging (MRI) studies on myocutaneous flaps utilized in orthopedic reconstructive surgery in 2006 demonstrated progressive atrophy of the muscle within these flaps over time. This atrophy was associated with fatty infiltration, which was evident as increased signal intensity on T1-weighted MRI sequences [5].

A Korean study conducted between 2009 and 2017 observed a decrease in the muscle component of the latissimus dorsi myocutaneous flap by ~24% between 6 months and 2 years postoperatively. Beyond the third postoperative year, the rate of muscle volume reduction stabilized to match that of native muscle, while the fat component of the flap remained relatively stable [6]. Similarly, a Japan study conducted in 2020 analysing free rectus abdominis myocutaneous flaps used in maxillary reconstruction found a reduction in muscle volume by <40% over time, with no significant changes in fat volume [7].

The observed muscle loss is likely attributable to denervation injury. Following denervation, muscle fibers undergo progressive atrophy and degeneration, and are subsequently replaced by fat and fibrous connective tissue [8].

Postoperative chemotherapy is another factor known to exacerbate muscle atrophy. A previous study in Korea documented a significant reduction in latissimus dorsi muscle volume after chemotherapy compared to immediate postoperative measurements [9]. However, in the present case, there was no documentation indicating that the patient underwent postoperative chemotherapy.

A further study conducted in Korea between 2007 and 2010 evaluated volume changes in TRAM flaps over two years using CT imaging. This study demonstrated a decrease in flap volume over time [10]. Unfortunately, due to a lack of immediate postoperative records for this patient from 2003, it was not possible to analyze longitudinal changes in overall breast volume for this case.

These findings collectively underscore the long-term morphological changes in myocutaneous flaps and the multifactorial etiology of muscle atrophy in such reconstructions.

## Conclusion

This report shows a case of a p-TRAM flap fat attenuation and muscle loss after 21 years with histopathological evidence. This provides evidence to confirm our current understanding of the phenomenon, which hitherto has been based predominantly on imaging studies.

## Data Availability

The raw datasets generated within this study are available from the corresponding author on reasonable request.
